# Interdisciplinary Multidimensional Assessment of Transthyretin Amyloidosis before and after Tafamidis

**DOI:** 10.3390/life13122305

**Published:** 2023-12-07

**Authors:** Manuela Pennisi, Giuseppe Lanza, Girolamo Aurelio Vitello, Denise Cristiana Faro, Francesco Fisicaro, Francesco Cappellani, Rita Bella, Ines Paola Monte

**Affiliations:** 1 Department of Biomedical and Biotechnological Sciences, University of Catania, Via Santa Sofia 89, 95123 Catania, Italy; manuela.pennisi@unict.it (M.P.); drfrancescofisicaro@gmail.com (F.F.); 2 Department of Surgery and Medical-Surgical Specialties, University of Catania, Via Santa Sofia 78, 95123 Catania, Italy; denisefaro88@gmail.com (D.C.F.); inemonte@unict.it (I.P.M.); 3 Oasi Research Institute-IRCCS, Via Conte Ruggero 73, 94018 Troina, Italy; avitello@oasi.en.it; 4 Ophthalmology Unit, Policlinico University Hospital “G. Rodolico-San Marco”, 95123 Catania, Italy; francescocappellani@hotmail.com; 5 Department of Medical, Surgical, and Advanced Technologies “G.F. Ingrassia”, University of Catania, Via Santa Sofia 87, 95123 Catania, Italy; rbella@unict.it

**Keywords:** amyloidosis, diagnosis, multidisciplinary, cardiomyopathy, cortical excitability, cerebral hemodynamics

## Abstract

Background: Clinically, there is considerable heterogeneity in the presentation of transthyretin amyloidosis (ATTR), which ranges from primarily cardiac and primarily neurologic to mixed disease, among other manifestations. Because of this complex presentation, the diagnosis and management of patients with ATTR are often challenging and should be performed in interdisciplinary centers specialized in amyloidosis. Here, we aimed to increase awareness of ATTR detection and pathophysiology through a multidimensional multiorgan approach. Case report: We reported on a 60-year-old man with wild-type ATTR who underwent a number of both basic and advanced cardiological and neurological investigations at baseline and after a treatment period with the TTR tetramer stabilizer, tafamidis. Several findings are provided here, some of which might be considered instrumental correlates of the patient’s clinical improvement after therapy. Conclusions: Adequate awareness and prompt recognition of ATTR support early diagnosis and faster access to therapies, thereby slowing the progression and improving the prognosis. The need for a multidisciplinary alliance between specialists and the opportunity to perform, at least in selected cases, a set of specific examinations for a detailed assessment of ATTR patients can also provide valuable insights into the physiopathology and response to therapy of a disease as complex and intriguing as ATTR.

## 1. Introduction

Amyloidoses are a wide group of proteotoxic diseases caused by the aggregation of specific proteins. Transthyretin (TTR) is an amyloidogenic protein in humans. Variant TTR deposition causes autosomal dominant hereditary TTR amyloidosis (ATTR), whereas wild-type ATTR (wtATTR) deposition leads to an acquired amyloid-related disease, i.e., the senile systemic amyloidosis, which typically presents later than the hereditary form [1].

ATTR is still considered a rare disease, although diagnostic progress indicates that there are many more patients than previously thought. Furthermore, wtATTR is a relatively common aging-related disorder, with postmortem studies indicating that more than 10% of people over the age of 80 may have TTR deposition [2]. While liver transplantation markedly improves the prognosis in familial amyloid polyneuropathy (FAP), a large proportion of patients are not suitable transplant candidates because of age or advanced disease. In addition, transplantation is not a viable option for familial amyloid cardiomyopathy, familial leptomeningeal amyloidosis, and wtATTR [1]. Recently, the molecular and biochemical pathogenesis of ATTR has been clarified, thus paving the way for novel disease-modifying treatments. Among them, the TTR tetramer stabilizer tafamidis has been associated with a lower risk of mortality and heart failure exacerbations in cardiac amyloidosis (CA) patients, as shown by several studies, including the ATTR-ACT trial, which showed a low risk of all-cause death, heart transplant, heart assist device implantation, and heart failure exacerbations or hospitalizations [3,4]. Although tafamidis cannot stop the progression of CA, tafamidis is able to slow it in some cases, particularly in the early-stage cases, and it is associated with reduced deterioration in the left ventricular ejection fraction (LVEF), especially in those with wtATTR. Additionally, studies have highlighted that tafamidis delays both structural and functional changes in the left ventricle [5,6], which is also supported by a very recent meta-analysis, indicating no significant LVEF decrease after treatment with tafamidis [7]. Lastly, long-term data suggest that tafamidis improves survival and quality of life, with significant benefits in terms of functional capacity [8].

Clinically, there is considerable heterogeneity in the presentation of ATTR, which ranges from primarily cardiac and primarily neurologic to mixed disease, among other manifestations (such as ocular, respiratory, gastrointestinal, and renal). Nevertheless, CA is still an underdiagnosed condition and an underestimated cause of heart failure and conduction abnormalities [9]. Nowadays, however, recognition of CA has improved, mainly due to the increased use of cardiac magnetic resonance imaging (MRI) and cardiac scintigraphy as non-invasive diagnostic tools, providing the early recognition of cardiac infiltration crucial for optimizing long-term prognosis [9]. Neurologically, typical signs of ATTR include a rapidly progressive sensory-motor and autonomic neuropathy, with carpal tunnel syndrome (CTS) often preceding other manifestations [1]. Although symptoms of progressive cardiomyopathy are usually prominent in the sporadic variant of wtATTR, neurological assessment of these patients often reveals a concomitant polyneuropathy.

Because of this complex presentation, the diagnosis and management of ATTR should be performed in interdisciplinary specialized centers. In this case report, we highlighted the importance of a close inter-specialty interaction to disentangle the complex multiorgan pathophysiology of this disorder and to determine the best management.

## 2. Case Report

A 60-year-old right-handed man, working as a teacher, came to our attention in June 2021 because of the onset, in the preceding months, of progressive asthenia, fatigability, and atypical chest pain. His past medical history included hypertension, dyslipidemia, right hip arthroplasty in 2005, and diverticulosis diagnosed approximately 20 years earlier, with periodic inflammation. He practiced regular physical activity and never smoked. At-home treatment included ranolazine 500 mg bis in die, carvedilol 12.5 mg bis in die, lysine acetylsalicylate 75 mg, perindopril + indapamide 10 + 12.5 mg, and simvastatin + ezetimibe 10 + 10 mg.

Since 2013, he experienced episodes of hypoesthesia in the right arm, which led to a neurological consultation, followed by a brain MRI. Several years later (February 2020), he started to complain of paresthesia in both hands, which later led to the diagnosis of bilateral carpal tunnel syndrome (CTS); however, these symptoms did not raise suspicion of amyloidosis. The initial cardiological manifestations appeared in June 2019; at that time, the patient underwent an exercise stress test for atypical chest pain, presyncope-like episodes, fatigue, and dyspnea after intense exercise. The results were doubtful for myocardial ischemia, and therefore, further investigation was required. The patient underwent coronary angiography, which revealed coronary ectasia and non-hemodynamically significant stenoses due to atheromatosis in the left coronary artery, left anterior descending artery, and first marginal branch artery, thus prompting a recommendation for medical therapy. Concomitantly, an echocardiogram revealed left ventricular hypertrophy with a septal thickness of 17 mm. However, again, this finding did not induce suspicion of CA, which instead was diagnosed (via I.P.M.) at our specialized Cardiological Rare Disease Center of the Policlinico University Hospital “G. Rodolico-San Marco” of Catania (Italy) approximately two years after the clinical onset (June 2021). An advanced echocardiographic exam showed concentric biventricular hypertrophy, normal contractile function (LVEF: 64%), signs of increased left ventricular filling pressure (E/e’ 11), elevated pulmonary artery systolic pressure (40 mmHg), and mild pericardial effusion. The global longitudinal strain was reduced (−15%), with an apical sparing pattern, suggesting amyloid infiltration.

Based on these findings, the patient started a comprehensive diagnostic work-up, eventually leading to the diagnosis of wtATTR. First, an electrocardiogram (ECG) showed sinus rhythm, a normal QRS complex, low voltages in peripheral leads, and ST-T-wave changes in inferior and lateral leads. Cardiac MRI revealed an LVEF of 67%, with normal kinetics but severe septal hypertrophy (maximal thickness: 22 mm, left ventricular mass index: 138 g/mq). Post-gadolinium imaging showed diffuse circumferential enhancement, predominantly subendocardially, with widespread enhancement in both atria, extracellular volume expansion, and slight pericardial effusion. The 99mTc-HDP bone scintigraphy displayed grade 2 Perugini uptake, consistent with CA (the Perugini grading scale [10] is a semi-quantitative method for scoring scintigraphy uptake in CA; grade 2: cardiac uptake intensity similar to that of rib uptake).

Among other investigations, serum and urine immunofixation were negative, with a normal free light chain kappa/lambda ratio (kappa: 13.5 mg/L, lambda: 15.7 mg/L, kappa/lambda ratio: 0.86). Laboratory findings included the N-terminal pro-b-type natriuretic peptide (NT-pro-BNP): 574 ng/L; high-sensitivity troponin I: 35 ng/L; creatinine: 1.19 mg/dL. The patient was graded as NYHA (New York Heart Association) IIa, with a Kansas City Cardiomyopathy Questionnaire score of 80, and classified as Stage 1 in the Gillmore staging system [11]. Both *TTR* gene mutation analysis and periumbilical fat aspiration (July 2021) were negative, thus excluding familial amyloidosis and confirming wtATTR as the final diagnosis.

Before starting specific treatment with tafamidis, a basic and advanced neurological assessment was carried out. First, the neurological examination result was normal, except for hypoexcitable reflexes at the lower limbs and hypoesthesia/paresthesia at the first three fingers. Then, a detailed study of nerve conduction (including H reflexes and F waves) was carried out (G.A.V.), which excluded polyneuropathy and detected a bilateral CTS, more severe on the left side. Needle electromyography, along with neurography data, showed chronic neurogenic damage compatible with bilateral cervical and lumbosacral radiculopathies (C5-C6-C7 and L4-L5-S1). The bilateral blink reflex was normal, thus excluding neuropathy or brainstem dysfunction, and a bilateral cutaneous reflex did not show signs of autonomic dysfunction. A brain MRI revealed signs of chronic cerebrovascular disease compatible with a Fazekas grade 1, i.e., periventricular white matter “caps” and punctuate foci in the deep white matter (the Fazekas scale is used to visually quantify the amount of white matter T2-weighted hyperintense lesions, usually attributed to chronic small vessel ischemia [12]), whereas a spine MRI showed multimetameric disk protrusions along the cervical tract, more severely at the C4–C5 and C5–C6 level. The patient was also screened by means of the Hamilton Depression Rating Scale (HDRS) and the Montreal Cognitive Assessment (MoCA): he scored 9 for HDRS (normal values: <7) and 24 for MoCA (normal values, adjusted for age and education: ≥26), which were compatible with mild depression and a degree of cognitive impairment (especially for the items probing the executive functions). Functional independence, indexed by the Activities of Daily Living (ADL) and the Instrumental ADL scales, was entirely normal.

To provide a more comprehensive neurological assessment, a bilateral study of the central motor conductivity (G.L.) and central sensory pathways (M.P.) was performed [13], both before and after therapy, by means of the motor-evoked potentials (MEPs) via transcranial magnetic stimulation (TMS) and the somatosensory evoked potentials (SEPs) via median nerve stimulation, respectively, as well as an in-depth evaluation of cerebral hemodynamics and vasomotor reactivity (CVR) through transcranial Doppler (TCD) sonography with the breath-holding test (a rapid and feasible maneuver for probing CVR) (R.B.). While basic MEP features (amplitude, latency, and central motor conduction time) were normal, the profile of cortical excitability [14], as indexed by the resting motor threshold (i.e., the lowest TMS intensity needed to evoke MEPs in the target muscle at rest when single-pulse stimuli are applied to the primary motor cortex) and the cortical silent period (i.e., suppression of the EMG activity evoked by a suprathreshold TMS applied over the contralateral motor cortex during a sustained voluntary contraction of the target muscle), decreased bilaterally from 41% to 36% and from 130 ms to 98 ms, respectively, thus hypothesizing a global pattern of cerebral hypoexcitability at baseline which ameliorated after treatment. Baseline SEPs showed prolonged central sensory conduction time and reduced amplitude responses at the upper limbs (probably due to the concomitant CTS), whereas SEPs showed normal findings after treatment. The TCD examination showed that all velocimetric parameters recorded from both middle cerebral artery decreased at follow-up, whereas the pulsatility and resistivity indexes (which are derived from flow parameters in ultrasound examinations, typically used to assess the resistance in a pulsatile vascular system [15]) tended to increase; no substantial change was noted from the basilar artery, as well as after the breath-holding test from all the arteries explored.

In October 2021, treatment with tafamidis was started, and the patient’s follow-up took place after approximately 6 months. The 6-Minute Walk Test improved from 490 m pre-tafamidis to 537 m in January 2022 and further increased to 600 m in June 2022. A dynamic ECG (January 2022) showed 46 premature ventricular contractions and 2 runs of ventricular and supraventricular ectopy. At the one-year follow-up, NT-pro-BNP decreased to 231 ng/L and troponin I to 20 ng/L, whereas creatinine remained stable at 1.15 mg/dL, along with the echocardiography findings. Cardiac MRI (June 2022) displayed stable LVEF (65%), interventricular septum thickness (20 mm), and myocardial mass, with a slight increase in T2, a median T1 of 1150 ms, and an extracellular volume of 53%. Overall, the echocardiographic findings were basically unchanged, except for the mild pericardial effusion, which was no longer evident. Meanwhile, the patient underwent bilateral surgery for his CTS. However, he still complained of paresthesia in both the upper (especially at the left fingers) and lower limbs (more at the left ankle), although the neurological examination remained within the normal limits. The HDRS score was borderline (7), whereas MoCA improved to a normal value (28). The patient was recently re-visited: he was still on tafamidis and appeared to be cardiologically stable, with improved effort tolerance, although symptoms of peripheral nervous system dysfunction persisted.

Informed consent was obtained from the subject involved in the study, which was conducted according to the guidelines of the Declaration of Helsinki of 1964 and later amendments and was approved by the Ethics Committee of the Azienda Ospedaliero-Universitaria Policlinico “G. Rodolico-San Marco” of Catania, Italy (approval code: 2019/0004003).

## 3. Discussion

ATTR is a clinically heterogeneous and potentially life-threatening disease that results from the deposition of insoluble amyloid fibrils in various organs and tissues, especially in cardiac and neuronal cells, causing progressive loss of function and disability. In this case report, we aimed to increase the awareness of ATTR detection through a multidimensional assessment and a multiorgan approach. Accordingly, we showed that adequate awareness and prompt recognition of ATTR support early diagnosis and faster access to therapies, thereby slowing the progression and improving the prognosis, as also demonstrated in other rare disorders characterized by multiorgan involvement [16,17].

Although the importance of a multidisciplinary approach with these patients is well-known, here we emphasized a “real-world” experience in the diagnosis and management of amyloidosis. First, we underlined the significant diagnostic delay (of approximately two years) which occurred before the patient came to our attention. Despite this, we were still able to successfully intercept him at an early stage; this timing also aligns with the consensus documents for the initiation of therapy (NYHA IIa, Gillmore Stage 1). Moreover, we reported a favorable response in terms of improved cardiac function and stabilization of the multiorgan progression. This outcome was supported by a detailed diagnostic cardiological and neurological work-up, as well as laboratory and biopsy exams.

As recently highlighted [18], a delayed diagnosis of CA is a significant issue, often due to a lack of awareness, misconceptions about diagnosis, the heterogeneity of CA presentation, and non-specific early symptoms. In this context, the Amyloidosis Expertise Center Utrecht has implemented an efficacious multidisciplinary clinical pathway: first, suspected patients are evaluated by a specialized team of cardiologists, hematologists, and neurologists. Then, they start a comprehensive assessment, including ECG, echocardiography, and lab tests (i.e., NT-proBNP, troponin, and estimated glomerular filtration rate, among others). In the case of clinical suspicion of CA, bone scintigraphy and screening for monoclonal protein are also performed; endomyocardial biopsy is only performed when a non-invasive diagnosis of CA cannot be established, whereas genetic screening and treatment plans are conducted as needed, along with shared decision-making and management, both based on regular follow-up and interdisciplinary consultations. This approach has shown promising results in terms of reduced diagnostic delay by an average time of 6 months and increased patient referral. Notably, the authors showed that the severity of CA at diagnosis improved, with fewer patients presenting with advanced disease (i.e., NYHA Class III) [18]. Similarly, Kittleson et al. [19] highlighted the diagnostic challenges of the ATTR, with many patients experiencing delays and consulting several physicians before receiving the correct diagnosis. This is often attributed to symptoms overlapping with other disorders and pleiomorphic clinical presentations, including musculoskeletal, neurological, gastrointestinal, ocular, and renal manifestations. Both studies underscore the need for a multidisciplinary collaboration, which involves, among others, the interpretation of monoclonal light chain tests, bone scintigraphy scans, and specific biopsies.

Until recently, in fact, ATTR was mainly considered a neurologic disease since peripheral neuropathic symptoms tended to predominate, especially in patients described in early reports (although, in the present case, peripheral polyneuropathy was excluded, both clinically and neurophysiologically). On the other hand, however, recent advances in diagnostic techniques and increased recognition have revealed the presence of patients with cardiomyopathy as a predominant feature; for this reason, ATTR is now primarily considered a cardiological disease. Nevertheless, recent studies have suggested that some patients with wtATTR have presented tenosynovial tissue complications as the initial manifestation, particularly CTS not otherwise explained [20,21] (as in the present report), thus also requiring awareness among orthopedists. Notwithstanding the limitations related to the single-case report design, however, the objective of this report was not to provide a reminder that sometimes “the third party enjoys between the two litigants” but to stress the need for a multidisciplinary alliance between specialists and the opportunity to perform, at least in selected cases, a set of specific examinations for a detailed assessment of ATTR. Results from this report might indeed disclose hints towards a better understanding of the neurological involvement in ATTR, including changes in the profile of cortical excitability and cerebral hemodynamics, which seems to be positively influenced by the TTR tetramer stabilizer. In larger samples, this approach can provide more robust insights into the physiopathology and response to therapy.

**Figure 1:**
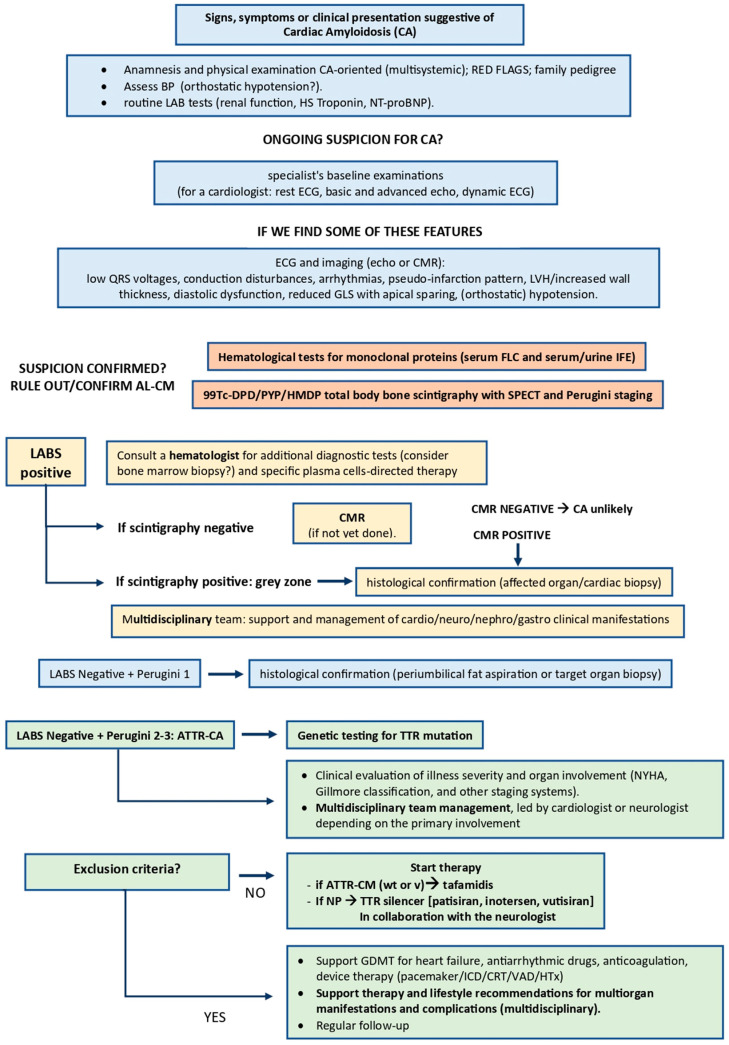
Flowchart summarizing the 2023 guidelines of the European Society of Cardiology (ESC) for cardiomyopathies, especially focusing on cardiac amyloidosis. Abbreviations: CA, cardiac amyloidosis; BP, blood pressure; HS, high sensitivity; NT-proBNP, N-terminal pro-b-type natriuretic peptide; ECG, electrocardiogram; echo, echocardiogram; CMR, cardiac magnetic resonance; LVH, left ventricular hypertrophy; GLS, global longitudinal strain; LABS, laboratory exams; AF, atrial fibrillation; VT, ventricular tachycardia; AL-CM, amyloid monoclonal immunoglobulin light chain cardiomyopathy; FLC, free-light chains; IFE, immunofixation electrophoresis; SPECT, single-photon emission computed tomography; ATTR-CM, transtiretin amyloid cardiomyopathy; NP, neuropathy; TTR, transthyretin; NYHA; New York Heart Association; wt, wild type; v, variant; GDMT, guideline-directed medical therapy; ICD, implantable cardioverter defibrillator; CRT, cardiac resynchronization therapy; VAD, ventricular assistance device; HTx, heart transplantation.

Interestingly, the CTS-related symptoms did not raise suspicion of amyloidosis; conversely, the cardiac features led to the performance of a cardiac MRI, which eventually confirmed the suspicion of CA. In this context, it is worth noting that the “red flags” from both the position statement of the European Society of Cardiology (ESC) Working Group on Myocardial and Pericardial Diseases [22] and the ESC 2023 guidelines on cardiomyopathies [23] are predominantly cardiological, except for the CTS. Based on the AHA/ACC as a reference document, hip arthroplasty and fatigue have been added to the list. Thus, in the present case, it can be stated that cardiac symptoms led to the suspicion of amyloidosis and the referral to a specialized center, while the non-cardiac features went unnoticed over the years. Table 1 lists the “red flags” identified in the literature compared to those observed in our patient, whereas Figure 1 shows a flowchart summarizing the ESC 2023 guidelines for cardiomyopathies, especially focusing on CA [18,19,22,23].

In conclusion, the case reported here reinforces the concept that, despite the severity of the disease and the risk of complications, an early and multidisciplinary diagnosis and treatment may lead to the maintenance of a normal daily life and an optimal quality of life. Moreover, we emphasize the importance of both cardiac and extracardiac manifestations of amyloidosis and the need for coordination and cooperation among all of the specialists involved in patient care. Further research is needed, and some unanswered questions still remain, particularly concerning equitable care and the full potential of such interdisciplinary collaboration, especially in the management of complex or doubtful cases.

## Figures and Tables

**Table 1:** Red flags for cardiac amyloidosis according to the European Society of Cardiology (ESC) 2023 guidelines for the management of cardiomyopathies compared to the findings observed in this study.

Red Flags for Cardiac Amyloidosis According to the ESC 2023 Guidelines for the Management of Cardiomyopathies	Red Flags Observed in This Patient
Left ventricular wall thickness ≥ 12 mm, + at least one of the following:	
Heart failure in ≥65 years old	
Aortic stenosis in ≥65 years old	
Hypotension or normotensive if previously hypertensive	
Sensory involvement, autonomic dysfunction	✔
Peripheral polyneuropathy	
Proteinuria	
Skin bruising	
Ruptured biceps tendon	
Bilateral carpal tunnel syndrome	✔
Subendocardial/transmural late gadolinium enhancement or increased extracellular volume	✔
Reduced longitudinal strain with apical sparing	✔
Decreased QRS voltage-to-mass ratio	✔
Pseudo Q waves on electrocardiogram	
Atrio-ventricular conduction disease	
Possible family history of transthyretin amyloidosis	
Chronically increased troponin levels (persistent low-level troponin elevation)	✔
Known multiple myeloma or monoclonal gammopathy of undetermined significance	

## Data Availability

Data presented in this study are available within the article.
